# Cardiovascular and renal outcomes with SGLT-2 inhibitors versus GLP-1 receptor agonists in patients with type 2 diabetes mellitus and chronic kidney disease: a systematic review and network meta-analysis

**DOI:** 10.1186/s12933-020-01197-z

**Published:** 2021-01-07

**Authors:** Takayuki Yamada, Mako Wakabayashi, Abhinav Bhalla, Nitin Chopra, Hirotaka Miyashita, Takahisa Mikami, Hiroki Ueyama, Tomohiro Fujisaki, Yusuke Saigusa, Takahiro Yamaji, Kengo Azushima, Shingo Urate, Toru Suzuki, Eriko Abe, Hiromichi Wakui, Kouichi Tamura

**Affiliations:** 1 Department of Medicine, Mount Sinai Beth Israel, Icahn School of Medicine at Mount Sinai, New York, NY USA; 2 Department of Medical Science and Cardiorenal Medicine, Yokohama City University Graduate School of Medicine, 3 Chome-9 Fukuura, Kanazawa Ward, Yokohama, Kanagawa 236-0004 Japan; 3 Department of Medicine, Nippon Medical School Hospital, Tokyo, Japan; 4 Department of Neurology, Tufts Medical Center, Boston, MA USA; 5 Department of Medicine, Mount Sinai Morningside and West, Icahn School of Medicine at Mount Sinai, New York, NY USA; 6 Department of Biostatistics, Yokohama City University School of Medicine, Yokohama, Japan

**Keywords:** SGLT2 inhibitors, GLP-1 receptor agonist, Meta-analysis, Cardiovascular disease, Renal outcomes, Diabetes mellitus, Chronic kidney disease

## Abstract

**Background:**

Emerging evidence suggests that sodium-glucose cotransporter-2 (SGLT-2) inhibitors and glucagon-like peptide-1 receptor agonists (GLP-1 RAs) are associated with decreased risk of cardiovascular and renal events in type 2 diabetes mellitus (DM) patients. However, no study to date has compared the effect of SGLT-2 inhibitors with that of GLP-1 RAs in type 2 DM patients with chronic kidney disease (CKD). We herein investigated the benefits of SGLT-2 inhibitors and GLP-1 RAs in CKD patients.

**Methods:**

We performed a systematic literature search through November 2020. We selected randomized control trials that compared the risk of major adverse cardiovascular events (MACE) and a composite of renal outcomes. We performed a network meta-analysis to compare SGLT-2 inhibitors with GLP-1 RAs indirectly. Risk ratios (RRs) with corresponding 95% confidence intervals (CI) were synthesized.

**Results:**

Thirteen studies were selected with a total of 32,949 patients. SGLT-2 inhibitors led to a risk reduction in MACE and renal events (RR [95% CI]; 0.85 [0.75–0.96] and 0.68 [0.59–0.78], respectively). However, GLP-1 RAs did not reduce the risk of cardiovascular or renal adverse events (RR 0.91 [0.80–1.04] and 0.86 [0.72–1.03], respectively). Compared to GLP-1 RAs, SGLT-2 inhibitors did not demonstrate a significant difference in MACE (RR 0.94 [0.78–1.12]), while SGLT-2 inhibitors were associated with a lower risk of renal events compared to GLP-1 RAs (RR 0.79 [0.63–0.99]). A sensitivity analysis revealed that GLP-1 analogues significantly decreased MACE when compared to placebo treatment (RR 0.81 [0.69–0.95]), while exendin-4 analogues did not (RR 1.03 [0.88–1.20]).

**Conclusions:**

In patients with type 2 DM and CKD, SGLT-2 inhibitors were associated with a decreased risk of cardiovascular and renal events, but GLP-1 RAs were not. SGLT-2 inhibitors significantly decreased the risk of renal events compared to GLP-1 RAs. Among GLP-1 RAs, GLP-1 analogues showed a positive impact on cardiovascular and renal outcomes, while exendin-4 analogues did not.

## Background

Diabetes mellitus (DM) is a major public health problem with a high prevalence. The International Diabetes Federation estimated that 351.7 million people of working age (20–64 years) had DM in 2019, and this number is expected to increase to 417.3 million by 2030 [1]. Type 2 DM is the leading cause of chronic kidney disease (CKD), accounting for roughly 36% of adult CKD in the United States [2]. CKD with DM can progress to end-stage renal disease (ESRD) [3], which confers a poor overall prognosis. Moreover, type 2 DM and CKD increase the risk of cardiovascular disease [4, 5]. Therefore, the prevention of CKD progression and cardiovascular events is essential for the management of patients with type 2 DM and CKD.

Sodium-glucose cotransporter-2 (SGLT-2) inhibitors are a new class of glucose-lowering agents. SGLT-2 inhibitors function through reducing renal tubular glucose reabsorption, thereby lowering blood glucose without stimulating insulin release. Several large cohort studies and randomized controlled trials (RCTs) have demonstrated favorable cardiovascular and renal outcomes associated with SGLT-2 inhibitors [6–10]. Moreover, recent RCTs revealed that SGLT-2 inhibitors are associated with favorable cardiovascular outcomes in CKD patients [11, 12].

Glucagon-like peptide-1 receptor agonists (GLP-1 RAs) decrease hemoglobin A1c by both stimulating glucose-dependent insulin secretion and reducing glucagon secretion [13]. GLP-1 RAs are known to reduce blood pressure (BP) [14] and body weight [15]. Large cohort studies and meta-analyses of RCTs demonstrated that GLP-1 RAs improve cardiovascular outcomes [16–20] The American Diabetes Association (ADA) recommends SGLT-2 inhibitors or GLP-1 RAs in type 2 DM patients who have atherosclerotic cardiovascular disease or kidney disease [21]. However, it remains unclear if GLP-1 RAs are beneficial to type 2 DM patients with CKD as well.

Several studies have investigated the impact of these novel agents on cardiovascular disease prevention; they show that SGLT-2 inhibitors and GLP-1 are comparable in their ability to decrease cardiovascular and renal events [18, 22]. However, no study has compared the effect of SGLT-2 inhibitors on renal and cardiovascular diseases with that of GLP-1 RAs in CKD patients, who are at a high risk of morbidity. We herein investigate the benefits of SGLT-2 inhibitors and GLP-1 RAs in CKD patients by network meta-analysis.

## Methods

### Literature search

The search strategy was conducted in accordance with the PRISMA (Preferred Reporting Items for Systematic Reviews and Meta-Analyses) extension statement for network meta-analysis [23, 24]. We performed a systematic search of PubMed, Medline, EMBASE, and the Cochrane Library from inception to November 20, 2020. The following keywords were applied: (“sodium-glucose cotransporter-2 inhibitors” [MeSH] OR “SGLT-2 inhibitor” OR SGLT-2 OR “empagliflozin” OR “dapagliflozin” OR “canagliflozin” OR “luseogliflozin” OR “ertugliflozin”) OR (GLP-1 OR “glucagon-like peptide-1 receptor “[MeSH] OR “glucagon-like peptide-1 receptor agonist” OR “exenatide” OR “liraglutide” OR “lixisenatide” OR “semaglutide” OR “dulaglutide” OR “albiglutide”) AND (“diabetes mellitus, type 2” [MeSH] OR “diabetes mellitus type 2” OR “type 2 diabetes mellitus” OR DM or diabetes) AND (“renal insufficiency, chronic” [MeSH] or CKD or “chronic kidney disease” or “kidney disease” or “kidney failure” or CKF or “chronic kidney failure” or “renal failure” or CRF or CRD or “chronic renal disease”) AND (“myocardial infarction” [MeSH] OR MI OR “coronary artery disease” [MeSH] OR “cardiovascular disease” [MeSH] OR “cerebrovascular disorders” [MeSH] OR “stroke” [MeSH] OR CVA OR “cerebrovascular accident” OR MACE OR “major adverse cardiovascular event” OR “death” OR mortality [MeSH] OR “all-cause mortality” OR “cardiovascular mortality” OR “heart failure” OR “end stage renal disease” OR “renal failure” OR “kidney failure” OR ESRD OR “renal death” OR “albuminuria” OR “urine albumin” OR “proteinuria” OR “urine protein”).

We restricted the search to human studies. Reference lists included in meta-analysis studies were reviewed to minimize missing relevant studies. Two independent and blinded authors (TY and AB) reviewed the search results separately to select studies based on inclusion and exclusion criteria. When a consensus was not reached between the two authors, a third author (MW) was consulted to reach a decision.

### Study selection

Studies were selected if they met the following criteria: (1) they were published in peer-reviewed journals; (2) they included adult patients (≥ 18 years old) with type 2 DM; (3) they were RCTs that compared SGLT-2 inhibitors or GLP-1 RAs with a placebo; (4) they compared the risk of major adverse cardiovascular events (MACE) between treatment and placebo groups; (5) they compared the risk of renal outcomes; and (6) they showed an incidence of MACE and a composite of renal outcomes in patients with CKD (defined as estimated glomerular filtration rate (eGFR) < 60 ml/min/1.73 m^2^). There was no restriction on publication language. Studies were excluded if (1) they included non-human subjects and (2) there was insufficient data for estimating the risk ratio (RR) even after contacting the authors.

### Outcomes

The primary efficacy outcome of this analysis was 3-point MACE (MACE-3), including cardiovascular death, myocardial infarction (MI), and stroke. The secondary outcome was a composite of renal outcomes, including ESRD, a decline in kidney function (such as worsening of eGFR or increasing creatinine), albuminuria, and renal or cardiovascular death. For studies reporting multiple renal outcomes, renal outcomes without albuminuria were prioritized for consistency.

### Data extraction and quality assessment

All data from eligible studies were abstracted independently by two investigators (TY and AB). Any conflicts in data extraction or quality assessment were resolved by a third reviewer (MW). For each study, data regarding the incidence of MACE and a composite of renal outcomes in each group were abstracted. We used the Cochrane risk of bias assessment to explore sources of bias in the RCTs included in this analysis [25]. Applying this tool, we evaluated the risk of bias in random sequence generation, allocation concealment, the blinding of participants and researchers, the blinding of outcome assessments, selective reporting, incomplete outcome data, and other metrics.

### Statistical analysis

We performed a network meta-analysis using the “netmeta” package (version 1.1-0) and R programming language (R Foundation for Statistical Computing, Vienna, Austria). The RRs and 95% confidence intervals (CIs) were estimated using Mantel–Haenszel methods. A random-effects model was used for the analysis. Heterogeneity was assessed by the probability value of the *I*^2^ variable [26, 27]. Heterogeneity was considered to be low, moderate, or high if I^2^ was 25%, 50%, or 75%, respectively.

## Results

### Literature search and included studies

**Fig. 1:**
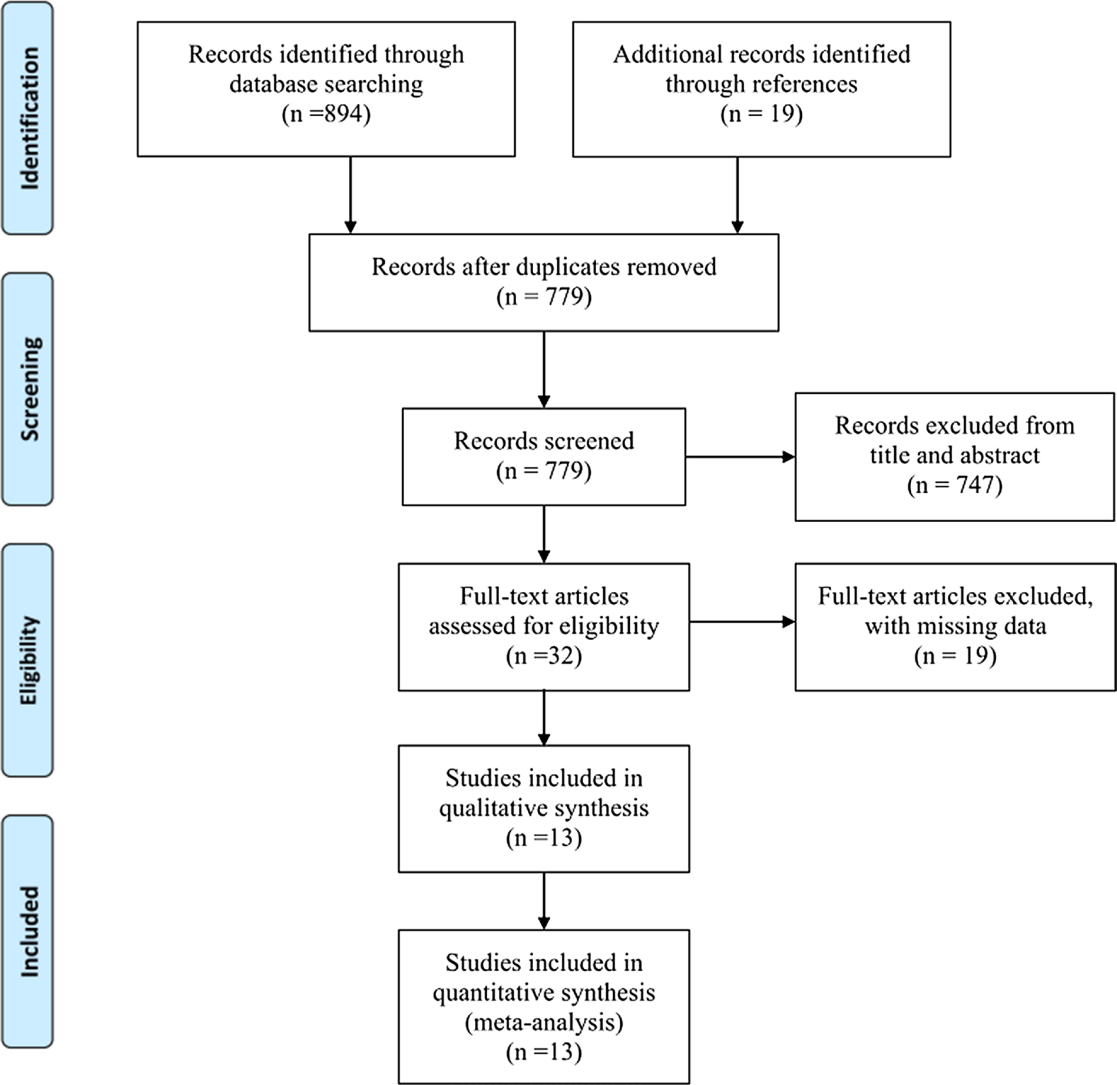
Flow diagram for study selection

A diagram of the study selection is shown in Fig. 1. Initially, a total of 894 studies were obtained in the primary search from databases, and nineteen additional studies were identified through references. We removed 134 duplicate studies; 779 studies were screened. By screening titles and abstracts, 747 papers were excluded because they did not meet the inclusion criteria. By assessing full-text articles, nineteen additional studies were excluded due to missing data. Finally, thirteen studies published up to November 20, 2020, were selected for our meta-analysis according to the inclusion criteria [12, 28–39]. Out of thirteen studies, six studies were RCTs that compared SGLT-2 inhibitors (Canagliflozin [12, 29], Dapagliflozin [31], Empagliflozin [30], Ertugliflozin [28], and Sotagliflozin [32]) with placebo; seven studies compared GLP-1 RAs (Albiglutide [34], Dulaglutide weekly [33], Exenatide weekly [35], Liraglutide [37], Lixisenatide [39], Semaglutide subcutaneously weekly [38], and Semaglutide oral [36]) with placebo. The pooled population consisted of 20,106 patients in SGLT-2 inhibitor studies (10,716 in the group treated with SGLT-2 inhibitors and 9390 in the control group) and 12,843 patients in GLP-1 RA studies (6364 in the group treated with GLP-1 RAs and 6479 in the control group). Hemoglobin A1c ranged from 6.5 to 12%. eGFR ranged from 15 to 59 ml/min/1.73 m^2^ in one study [33], 25 to 59 ml/min/1.73 m^2^ in one study, [32] and from 30 to 59 ml/min/1.73 m^2^ in other studies, with two studies that not mentioned their lower limit [31, 38]. The median length of follow-up ranged from 16.0 months to 50.4 months in SGLT-2 inhibitor studies and 19.2 months to 64.8 months in GLP-1 RA studies. All studies defined MACE-3 as a composite outcome comprised of cardiovascular death, myocardial infarction, and stroke, except ELIXA, [39] which also includes unstable angina. For renal outcomes, all studies included ESRD; five studies included renal death; [28–30, 33, 35] two studies included renal or cardiovascular death; [12, 31] one study defined reduced kidney function as a decrease in eGFR ≥ 30% [33], three as a decrease in eGFR ≥ 40% [29, 31, 35], one as a decrease in eGFR ≥ 50% [11], and four as a doubling of creatinine [12, 28, 30, 37].

### Study characteristics and quality assessment

**Fig. 2:**
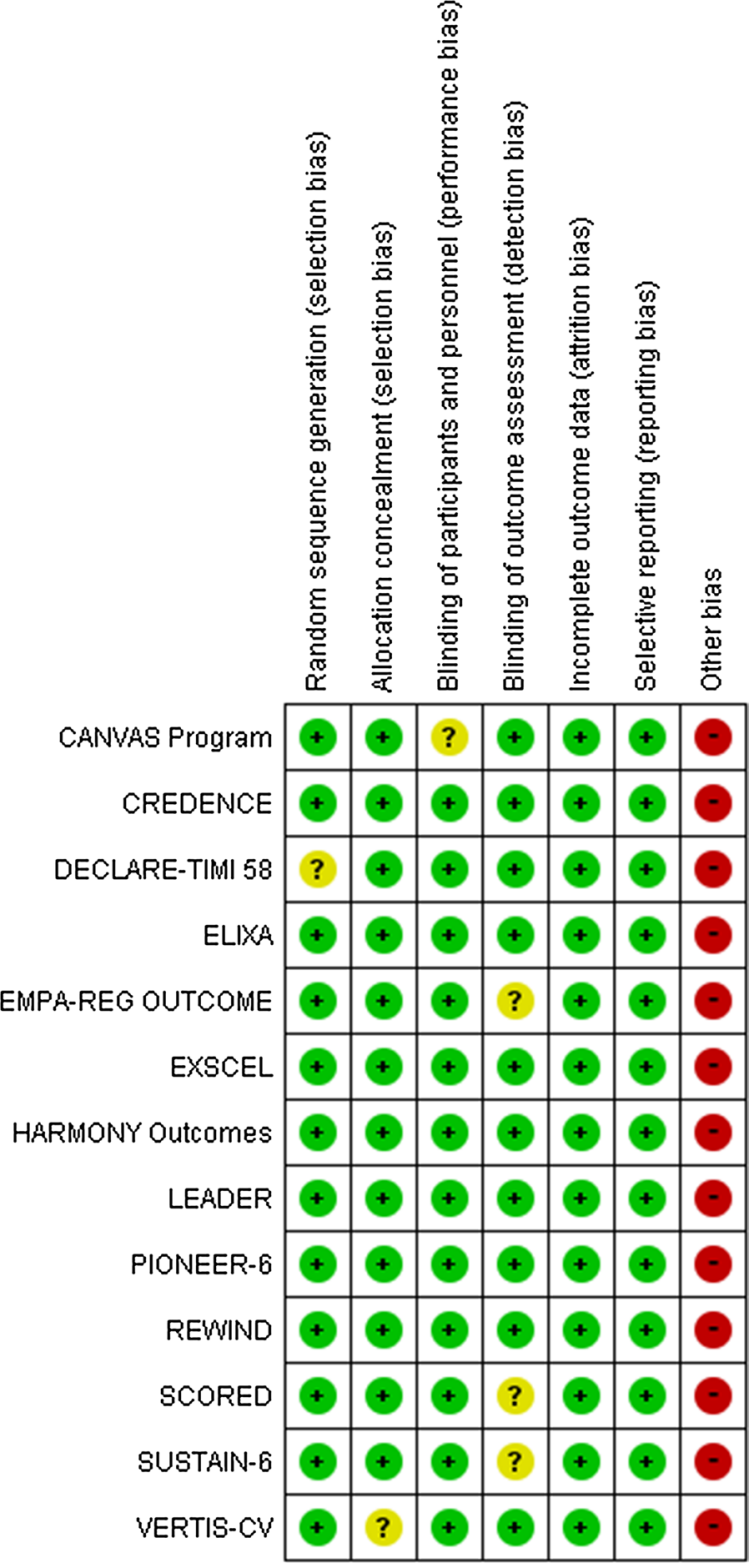
Quality assessment (Cochrane risk of bias tool) for included RCTs. *RCT* randomized control study

The definitions of terms, including a composite of renal outcomes and characteristics of the included studies, are listed in Table 1. Table 2 highlights the demographics of included studies. All studies defined CKD as eGFR < 60 ml/min/1.73 m^2^. The quality evaluation of the included studies is shown in Fig. 2. Subclasses of GLP-1 RAs (exendin-4 analogues or human GLP-1 RA analogues) in included studies are outlined in Additional file 1: Table S1.

**Table 1:** Definitions of terms in included studies

Study	Study design	Setting	Drug dose (mg/day)	Median follow up (months)	eGFR (ml/min/1.73 m^2^)	Range of Hgb A1c	Primary outcome	Definition of renal outcomes
SGLT2i vs placebo								
CANVAS Program	RCT	Multinational	Canagliflozin 300/100	29.0	30–59	7.0–10.5	MACE	≥ 40% eGFR decline, ESRD, renal death
CREDENCE	RCT	Multinational	Canagliflozin 100	31.4	30–59	6.5–12.0	Renal outcomes	Doubing creatinine, ESRD, renal or CV death
DECLARE-TIMI 58	RCT	Multinational	Dapagliflozin 10	50.4	<60	6.5–12.0	MACE	≥ 40% eGFR decline, ESRD, renal or CVdeath
EMPA-REG OUTCOME	RCT	Multinational	Empagliflozin 10/25	37.2	30–59	7.0–9.0	MACE	Doubling creatinine, ESRD, renal death
SCORED	RCT	Multinational	Sotagliflozin 400	16.0	25–60	> 7.0	MACE	≥ 50% eGFR decline, ESRD
VERTIS-CV	RCT	Multinational	Ertugliflozin 5/15	36.0	30–59	7.0–10.5	MACE	Doubing creatinine, ESRD, renal death
GLP-1 RA vs placebo								
ELIXA	RCT	Multinational	Lixisenatide 20 mcg	25.2	30–59	5.5–11.0	MACE (including unstable angina)	N/A
EXSCEL	RCT	Multinational	Exenatide 2 (weekly)	38.4	30–59	6.5–10	MACE	≥ 40% EGFR decline, ESRD, renal death
HARMONY Outcomes	RCT	Multinational	Albiglutide 30/50	19.2	30–59	> 7.0	MACE	N/A
LEADER	RCT	Multinational	Liraglutide 1.8	45.6	30–59	> 7.0	MACE	Doubling of serum creatinine, ESRD
PIONEER-6	RCT	Multinational	Semaglutide 14 (oral)	15.9	30–59	N/A	MACE	N/A
REWIND	RCT	Multinational	Dulaglutide 1.5 (weekly)	64.8	15–59	< 9.5	MACE	≥ 30% eGFR decline, ESRD, renal death
SUSTAIN-6	RCT	Multinational	Semaglutide 0.5/1 (weekly)	25.2	<60	> 7.0	MACE	N/A

*SGLT2i* sodium-glucose cotransporter-2, *GLP-1 RA* glucagon-like peptide-1 receptor agonist, *RCT* randomized control study, *eGFR* estimated glomerular filtration rate, *Hgb A1c* hemoglobin A1c, *ESRD* end-stage renal disease, *MACE* major adverse cardiovascular events, *RCT* randomized control trial, *CV* cardiovascular

**Table 2:** Baseline characteristics of included studies in patients with GFR < 60 ml/min/1.73 m^2^

Study	Number of patients (GFR < 60)	Age	Male (%)	BMI	sBP (mmHg)	dBP (mm Hg)	HgbA1c (%)	GFR < 30 (%)	GFR 30-45 (%)	GFR 45-60 (%)	Median GFR in CKD	UACR (mg/g)	Noormo-albuminuria (%)	Microalbuminuria (%)	Macroalbuminuria (%)
SGLT2i vs placebo															
CANVAS Program	2039	67.6	58.2	32.3	138	75	8.2	0.0	27.2	72.8	49.1	24.4	55.4	N/A	N/A
CREDENCE	2592	63.8	66.5	31.3	140	78	8.2	0.0	45.3	48.1	45.1	1012	N/A	N/A	N/A
DECLARE-TIMI 58	1265	67.3	64.3	34.5	134	75	8.2	N/A	N/A	N/A	51.4	N/A	55.6	30.9	13.5
EMPA-REG OUTCOME	1819	67.1	72.8	30.6	138	76	8.1	0	31.3	68.7	N/A	N/A	47.7	33.9	18.4
SCORED	10,584	69.0	55.1	31.8	138	78	8.3	7.7	43.9	48.3	44.5	74.5	35.1	33.9	31.0
VERTIS-CV	1807	N/A	N/A	N/A	N/A	N/A	N/A	N/A	N/A	N/A	N/A	N/A	N/A	N/A	N/A
Weighted average (SGLT-2 inhibitors)		67.8	59.5	31.9	137.9	77.2	8.2		40.8	53.4			40.8	33.6	27.7
GLP-1 RA vs placebo															
ELIXA	1399	N/A	N/A	N/A	N/A	N/A	N/A	N/A	N/A	N/A	N/A	N/A	59.7	25.5	14.8
EXSCEL	3177	66.5	57.1	32.8	N/A	N/A	8.1	0	28.0	72.0	49.2	N/A	71.4	19.6	9.0
HARMONY Outcomes	2222	N/A	N/A	N/A	N/A	N/A	N/A	N/A	N/A	N/A	N/A	N/A	N/A	N/A	N/A
LEADER	2158	67.3	61.3	32.7	136.4	75.1	8.6	10.4	29.0	60.6	45.6	49.5	47.6	29	23.4
PIONEER-6	856	N/A	N/A	N/A	N/A	N/A	N/A	N/A	N/A	N/A	N/A	N/A	N/A	N/A	N/A
REWIND	2199	N/A	N/A	N/A	N/A	N/A	N/A	N/A	N/A	N/A	N/A	N/A	N/A	N/A	N/A
SUSTAIN-6	832	N/A	N/A	N/A	N/A	N/A	N/A	N/A	N/A	N/A	N/A	N/A	N/A	N/A	N/A
Weighted average (GLP-1 RAs)		66.8	58.8	32.8			8.3		28.4	67.4			61.3	23.8	14.8

Normoalbuminuria, microlabuminuria and macroalbuminuria defined as < 30, 30–299, and ≥ 300 mg/g creatinine, respectively

*SGLT2i* sodium-glucose cotransporter-2 inhibitors, *GLP-1 RAs* glucagon-like peptide-1 receptor agonists, *GFR* glomerular filtration rate, *BMI* body mass index, *sBP* systolic blood pressure, *dBP* diastolic blood pressure, *Hgb A1c* hemoglobin A1c, *UACR* urine albumin-to-creatinine ratio, *N/A* not available

### Network meta-analysis of treatment groups

#### Mace-3

**Fig. 3:**
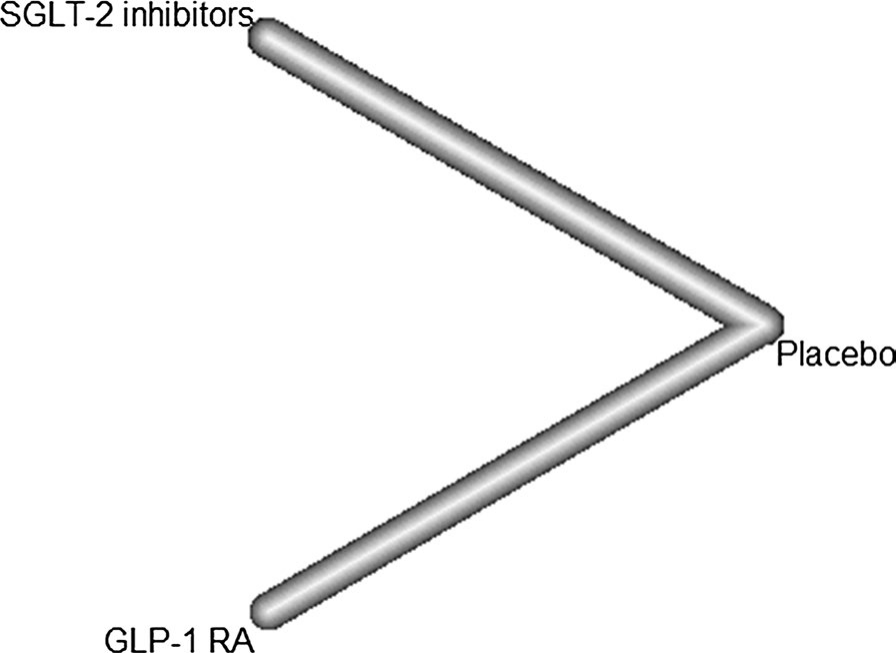
Network plot for MACE. *SGLT-2* sodium-glucose cotransporter 2, *GLP-1 RA* glucagon-like peptide-1 receptor agonist, *MACE* major adverse cardiovascular events

**Fig. 4:**
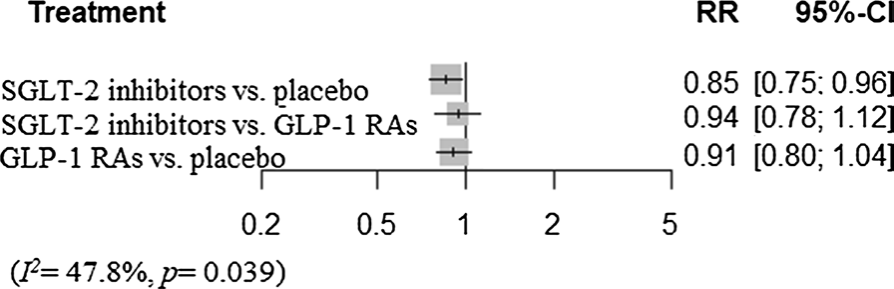
Network meta-analysis reporting risk ratio (RR) for MACE in CKD patients. *SGLT-2* sodium-glucose cotransporter 2, *GLP-1 RA* glucagon-like peptide-1 receptor agonist, *MACE* major adverse cardiovascular events, *CKD* chronic kidney disease

Network plots were shown in Fig. 3. SGLT-2 inhibitors were associated with a decreased risk of MACE-3 compared with placebo (RR [95% CI]; 0.85 [0.75–0.96]), but GLP-1 RAs were not (RR 0.91 [0.80–1.04]). Compared to GLP-1 RAs, SGLT-2 inhibitors did not show a significant difference in the risk of MACE-3 (RR 0.94 [0.78–1.12]) (Fig. 4). However, there was significant heterogeneity (*I*^*2*^= 47.8%, *p *= 0.039).

#### Renal outcomes

**Fig. 5:**
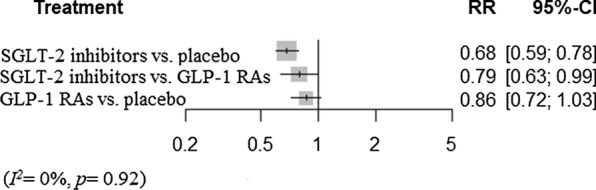
Network meta-analysis reporting risk ratio (RR) for renal outcomes in CKD patients. *SGLT-2* sodium-glucose cotransporter 2, *GLP-1 RA* glucagon-like peptide-1 receptor agonist, *CKD* chronic kidney disease

We also performed a network meta-analysis of the risk of renal events and found that SGLT-2 inhibitors significantly decreased renal events (RR 0.68 [0.59–0.78]), while the impact on renal events of GLP-1 RAs was not statistically significant (RR 0.86 [0.72–1.03]). SGLT-2 inhibitors were also associated with lower risk compared to GLP-1 RAs (RR 0.79 [0.63–0.99]) (Fig. 5). There was no heterogeneity (*I*^*2*^= 0%, *p *= 0.92).

### Sensitivity analyses

#### Mace-3

The results of sensitivity analyses are summarized in Table 3. First, we performed a sensitivity analysis for studies that defined MACE-3 as the primary outcome. Compared to placebo, SGLT-2 inhibitors had a tendency to decrease a risk of MACE-3 (RR 0.87 [0.76–1.00]), while GLP-1 RAs did not (RR 0.91 [0.79–1.04]). There was no significant difference between SLGT-2 inhibitors and GLP-1 RA (RR 0.96 [0.79–1.17]). There was significant heterogeneity (*I*^*2*^= 50.4%, *p *= 0.033).

**Table 3:** The summary of sensitivity analyses

Sensitivity analysis	Subjects						
	SGLT-2 inhibitors	GLP-1 RAs	Comparison	Risk ratio	95% CI	*I*^*2*^ *(%)*	*p value*
MACE-3							
MACE-3 as the primary outcome	17514	10644	SGLT-2 inhibitors vs. placebo	0.87	0.76–1.00	50.4	0.033
			GLP-1 RAs vs. placebo	0.91	0.79–1.04		
			SGLT-2 inhibitors vs. GLP-1 RAs	0.96	0.79–1.17		
Without ELIXA	20106	14007	SGLT-2 inhibitors vs. placebo	0.85	0.75–0.97	49.5	0.037
			GLP-1 RAs vs. placebo	0.88	0.76–1.03		
			SGLT-2 inhibitors vs. GLP-1 RAs	0.97	0.79–1.18		
Daily GLP-1 RAs	20106	6635	SGLT-2 inhibitors vs. placebo	0.84	0.75–0.96	47.7	0.053
			GLP-1 RAs vs. placebo	0.86	0.73–1.02		
			SGLT-2 inhibitors vs. GLP-1 RAs	0.99	0.80–1.21		
Weekly GLP-1 RAs	20106	4009	SGLT-2 inhibitors vs. placebo	0.85	0.76–0.95	31.8	0.19
			GLP-1 RAs vs. placebo	1.01	0.83–1.22		
			SGLT-2 inhibitors vs. GLP-1 RAs	0.85	0.68–1.05		
GLP-1 analogues	20106	6068	SGLT-2 inhibitors vs. placebo	0.85	0.76–0.95	30.6	0.17
			GLP-1 RAs vs. placebo	0.81	0.69–0.95		
			SGLT-2 inhibitors vs. GLP-1 RAs	1.05	0.86–1.27		
Exendin-4 analogues	20106	4576	SGLT-2 inhibitors vs. placebo	0.85	0.77–0.94	28.7	0.21
			GLP-1 RAs vs. placebo	1.03	0.88–1.20		
			SGLT-2 inhibitors vs. GLP-1 RAs	0.83	0.69–0.99		
Renal outcomes							
Renal endpoints as secondary outcomes	18583	7534	SGLT-2 inhibitors vs. placebo	0.67	0.55–0.83	0	0.86
			GLP-1 RAs vs. placebo	0.86	0.72–1.03		
			SGLT-2 inhibitors vs. GLP-1 RAs	0.78	0.59–1.03		
Macroalbuminuria	1505	7534	SGLT-2 inhibitors vs. placebo	N/A	N/A	0	0.73
			GLP-1 RAs vs. placebo	0.91	0.81–1.02		
			SGLT-2 inhibitors vs. GLP-1 RAs	N/A	N/A		
Weekly GLP-1 RAs	20088	5376	SGLT-2 inhibitors vs. placebo	0.65	0.55–0.78	0	0.73
			GLP-1 RAs vs. placebo	0.91	0.81–1.02		
			SGLT-2 inhibitors vs. GLP-1 RAs	0.71	0.58–0.89		
GLP-1 analogues	20088	4357	SGLT-2 inhibitors vs. placebo	0.68	0.59–0.78	0	0.94
			GLP-1 RAs vs. placebo	0.82	0.66–1.01		
			SGLT-2 inhibitors vs. GLP-1 RAs	0.83	0.65–1.07		

*SGLT2i* sodium-glucose cotransporter-2 inhibitors, *GLP-1 RAs* glucagon-like peptide-1 receptor agonists, *CI* confidence intervals, *MACE-3* 3-point major adverse cardiovascular event, *vs.* versus, *N/A* not available

Next, we conducted another sensitivity analysis excluding ELIXA, [39] as ELIXA includes unstable angina in its definition of adverse events in addition to MACE-3. The results were consistent: GLP-1 RAs did not show a significant difference when compared to placebo (RR 0.88 [0.76–1.03]) with significant heterogeneity (*I*^*2*^= 49.5%, *p *= 0.037). SGLT-2 inhibitors did not reduce MACE-3 significantly when compared to GLP-1 RAs (RR 0.97 [0.79–1.18]).

Third, we performed another analysis based on the frequency of GLP-1 RA dosing. Daily GLP-1 RAs tended to reduce the risk of MACE-3 when compared to placebo (RR 0.86 [0.73–1.02]), while weekly GLP-1 RAs did not (RR 1.01 [0.83–1.22]). GLP-1 RAs were comparable to SGLT-2 inhibitors (RR 0.99 [0.80–1.21] and 0.85 [0.68–1.05], respectively). Both analyses showed moderate heterogeneity (*I*^*2*^= 47.7% and 31.8%, respectively).

Lastly, we divided GLP-1 RAs into two subclasses, GLP-1 analogues and exendin-4 analogues, and found that GLP-1 analogues displayed significantly lower risk than placebo (RR 0.81 [0.69-0.95]), although there was moderate heterogeneity (*I*^*2*^= 30.6%, *p *= 0.17). The risk reduction between SGLT-2 inhibitors and GLP-1 analogues was similar (RR 1.05 [0.86-1.27]). Exendin-4 analogues, on the other hand, were not associated with a decreased risk of MACE-3 compared to placebo (RR 1.03 [0.88–1.20]), with moderate heterogeneity (*I*^*2*^= 28.7%, *p *= 0.21). SGLT-2 inhibitors were associated with significantly lower risk compared to exendin-4 analogues (RR 0.83 [0.69–0.99]).

#### Renal outcomes

Since most studies classify renal outcomes as secondary endpoints, we conducted a sensitivity analysis excluding the study classifying renal events as primary outcomes [12]. When compared to placebo, SGLT-2 inhibitors reduced the incidence of renal outcomes (RR 0.67 [0.55–0.83]). GLP-1 RAs did not show a statistical difference when compared to placebo (RR 0.86 [0.72–1.03]). SGLT-2 inhibitors tended to reduce the risk when compared to GLP-1 RAs (RR 0.78 [0.59–1.03]). No heterogeneity was appreciated (*I*^*2*^= 0%, *p *= 0.86).

Since GLP-1 RA studies included macroalbuminuria as a prespecified renal outcome, we performed a sensitivity analysis of studies that included macroalbuminuria as a renal outcome. The prespecified definitions of renal outcomes are shown in Additional file 1: Table S2. GLP-1 RAs were not associated with significantly reduced risk compared to placebo (RR 0.91 [0.81–1.02]). Only one SGLT-2 study included macroalbuminuria in renal outcomes.

Additional analysis based on GLP-1 RA frequency showed similar results. Weekly GLP-1 RAs did not decrease renal events compared to placebo (RR 0.91 [0.81-1.02]). There was no heterogeneity (*I*^*2*^= 0%, *p *= 0.73). SGLT-2 inhibitors were superior to weekly GLP-1 RAs (RR 0.71 [0.58–0.89]). Only one study investigated daily GLP-1 RAs.

We performed another analysis for GLP-1 analogues. GLP-1 analogues had a trend towards a reduction in renal events compared to placebo (RR 0.82 [0.66-1.01]) without heterogeneity (*I*^*2*^= 0%, *p *= 0.94). Compared to GLP-1 analogues, SGLT-2 inhibitors were associated with lower risk (RR 0.68 [0.59–0.78]). There was only one study that investigated renal risks for exendin-4 analogues.

#### Subgroup analysis

The results of subgroup analyses are summarized in Table 4. We conducted a subgroup analysis based on eGFR: 30-44 ml/min/1.73 m^2^ and 45-59 ml/min/1.73 m^2^. In a subgroup of eGFR: 30-44 ml/min/1.73 m^2^ patients, SGLT-2 inhibitors reduced MACE-3 significantly (RR 0.73 [0.54-0.97]), but GLP-1 RAs did not (RR 1.02 [0.78-1.33]). There was high heterogeneity (*I*^*2*^= 57.1%, *p *= 0.063). SGLT-2 inhibitors also showed beneficial effects on renal outcomes compared with placebo (RR 0.75 [0.62-0.91]), while GLP-1 RAs did not (RR 0.78 [0.46-1.32]). There was no heterogeneity (*I*^*2*^= 0%, *p *= 0.69). Compared to GLP-1 RAs, SGLT-2 inhibitors did not achieve a statistically significant difference (RR 0.96 [0.55–1.29]).

For patients with eGFR 45-59 ml/min/1.73 m^2^, both SGLT-2 inhibitors and GLP-1 RAs had a similar tendency to reduce MACE-3 compared to placebo (RR 0.82 [0.66-1.01] and 0.85 [0.71–1.03], respectively). There was moderate heterogeneity (*I*^*2*^= 44.8%, *p *= 0.12). In terms of renal outcomes, SGLT-2 inhibitors reduced renal outcomes (RR 0.61 [0.48-0.77), but GLP-1 RAs did not (RR 1.18 [0.76–1.84]). A comparison between SGLT-2 inhibitors and GLP-1 RAs was not statistically significant (RR 0.52 [0.31–0.85]). No heterogeneity was observed (*I*^*2*^= 0%, *p *= 0.52).

**Table 4:** The summary of subgroup analyses

Subgroup analysis	Subjects						
	SGLT-2 inhibitors	GLP-1 RAs	Comparison	Risk ratio	95% CI	*I*^*2*^ (%)	*p value*
MACE-3							
eGFR 30-44	2479	1934	SGLT-2 inhibitors vs. placebo	0.73	0.54–0.97	57.1	0.063
			GLP-1 RAs vs. placebo	1.02	0.78–1.33		
			SGLT-2 inhibitors vs. GLP-1 RAs	0.72	0.48–1.06		
eGFR 45-59	3974	4576	SGLT-2 inhibitors vs. placebo	0.82	0.66–1.01	44.8	0.12
			GLP-1 RAs vs. placebo	0.85	0.71–1.03		
			SGLT-2 inhibitors vs. GLP-1 RAs	0.96	0.72–1.27		
Renal outcomes							
eGFR 30-44	2420	889	SGLT-2 inhibitors vs. placebo	0.75	0.62–0.91	0	0.69
			GLP-1 RAs vs. placebo	0.78	0.46–1.32		
			SGLT-2 inhibitors vs. GLP-1 RAs	0.96	0.55–1.29		
eGFR 45-59	3976	2288	SGLT-2 inhibitors vs. placebo	0.61	0.48–0.77	0	0.52
			GLP-1 RAs vs. placebo	1.18	0.76–1.84		
			SGLT-2 inhibitors vs. GLP-1 RAs	0.52	0.31–0.85		

*SGLT2i* sodium-glucose cotransporter-2 inhibitors, *GLP-1 RAs* glucagon-like peptide-1 receptor agonists, *eGFR* estimated glomerular filtration rate, *CI* confidence intervals, *MACE-3* 3-point major adverse cardiovascular event, *vs.* versus, *N/A* not available

## Discussion

Our study revealed that SGLT-2 inhibitors decrease the risk of cardiovascular and renal events in type 2 DM patients with CKD, which is compatible with RCTs of CKD patients [11, 12]. On the other hand, GLP-1 RAs did not lead to significantly lower cardiovascular or renal endpoints, although they showed numerically better results. An indirect comparison of SGLT-2 inhibitors with GLP-1 RAs revealed that SGLT-2 inhibitors significantly decreased the risk of renal outcomes. Sensitivity analyses showed a similar tendency. Interestingly, a sensitivity analysis among GLP-1 RA subclasses revealed that GLP-1 analogues significantly reduced MACE-3 and renal events, while exendin-4 analogues did not.

Several mechanisms have been proposed for the positive impact of SGLT-2 inhibitors. First, SGLT-2 inhibitors have mild natriuretic and diuretic effects [40]. Recent RCTs reveal that in patients with heart failure with reduced ejection fraction, SGLT-2 inhibitors were associated with a lower risk of cardiovascular death or hospitalization for heart failure [41, 42]. This can be at least partially attributed to diuretic effects, which lead to BP reduction and thereby confer cardiovascular and renoprotective benefits. Second, SGLT-2 inhibitors mitigate low-grade inflammation. SGLT-2 inhibitors prevent glucose entry into proximal tubular cells, which limits glucotoxicity, potentially leading to less oxidative stress [43]. Third, SGLT-2 inhibitors block sodium reuptake in the proximal tubule; an increased delivery of sodium to the macula densa leads to afferent arteriolar constriction and a reduction in intraglomerular pressure [44, 45].

Since SGLT-2 inhibitors antagonize glucose reabsorption in the renal tubule, we can anticipate that the effect of SGLT-2 inhibitors is dependent on eGFR. However, a pooled analysis of clinical trials revealed that SGLT-2 inhibitors decrease body weight, BP, and albuminuria regardless of eGFR, although glucose-lowering effects decreased as eGFR declined [11, 46]. Our study also revealed beneficial effects of SGLT-2 inhibitors in both eGFR 30-44 ml/min/1.73 m^2^ and 45–59 ml/min/1.73 m^2^ groups.

In addition to glycemic control, several mechanisms explain the beneficial effects of GLP-1 RAs. First, GLP-1 RAs lead to BP reduction, [14] which can be attributed to natriuresis [47]. Other possible mechanisms are the reduction of reactive oxygen species and inflammation [48, 49] and improvement of endothelial function [50]. However, our study did not find a significant difference between GLP-1 RAs and placebo. A subgroup analysis of a prior meta-analysis by Kristensen et al. [17] also revealed that GLP-1 RAs were not associated with a significantly reduced risk of MACE, although the RR was 0.88. Our sensitivity analysis among GLP-1 RAs drug subclasses revealed a beneficial effect of GLP-1 analogues, while exendin-4 analogues were not. Zelniker et al. revealed a similar tendency [18]. There are several differences between these two subclasses. First, exendin-4 analogues are metabolized and eliminated by the kidneys. GLP-1 analogues, in contrast, are endogenously metabolized [51]. Second, exendin-4 analogues are resistant to inactivation by dipeptidyl peptidase-4. Conversely, GLP-1 analogues can be partially metabolized to the metabolite, which could have an additional cardioprotective effect [52]. As far as we know, there are no studies that evaluate the difference in the cardiovascular benefits between these subclasses. Further investigation is warranted to explore the differences among GLP-1 RAs subclasses.

The major strength of our analysis is that this is the first study that investigates the effect of SGLT-2 inhibitors and GLP-1 RAs on cardiovascular and renal events in type 2 DM patients with CKD [53]. The ADA preferably recommends SGLT-2 inhibitors over GLP-1 RAs in CKD patients; [21] our study supports their recommendations, with additional evidence that GLP-1 analogues can be an alternative option. Our large sample size was another strength of our analysis, allowing us to tease out statistically significant differences among interventions.

Our meta-analysis has several limitations. First, there is still a concern that CKD patients may not be fully randomized since the studies included are subgroup analyses of RCTs. Second, high heterogeneity was observed, and this heterogeneity persisted in most sensitivity and subgroup analyses. As shown in Table 2, the proportions of advanced CKD stages and macroalbuminuria are noticeably different. Population variety among studies may be a source of this heterogeneity. The results should be interpreted with caution. Third, the patients included in each trial may have achieved different levels of hemoglobin A1c, which may be subject to bias as well. Fourth, definitions of renal outcomes are not consistent across studies, though we prioritized renal outcomes without macroalbuminuria in an attempt to reduce inconsistency. Since most GLP-1 RAs studies included macroalbuminuria as a prespecified renal outcome, analyzing renal outcomes without macroalbuminuria may lead to bias. Lastly, we defined CKD as eGFR < 60 ml/min/1.73 m^2^ and excluded patients with albuminuria. Therefore, we were not able to investigate the effects of SGLT-2 inhibitors and GLP-1 RAs for those who have albuminuria and eGFR > 60 ml/min/1.73 m^2^. Ongoing RCTs, including EMPA-KIDNEY, SOUL, and FLOW, will provide better insight into the cardiorenal effects of SGLT-2 inhibitors and GLP-1 RAs [54–56]. Further research, including head-to-head comparison, is warranted to explore the effects of SGLT-2 inhibitors and GLP-1 RAs in CKD patients [57].

## Conclusion

In patients with type 2 DM and CKD defined as eGFR < 60 ml/min/1.73 m^2^, SGLT-2 inhibitors were associated with decreased risk of cardiovascular and renal events. GLP-1 RAs did not lead to significantly lower cardiovascular or renal endpoints, although they showed numerically better results. SGLT-2 inhibitors significantly decrease the risk of renal events compared to GLP-1 RAs. Sensitivity analyses were consistent. Among GLP-1 RAs, GLP-1 analogues showed a positive impact, while exendin-4 analogues did not.

## Supplementary information

**Additional file 1.** Supplementary tables.

## Data Availability

All data generated or analyzed during this study are included in this published article.

## Abbreviations

DM: Diabetes mellitus
CKD: Chronic kidney disease
ESRD: End-stage renal disease
SGLT-2: Sodium-glucose cotransporter-2
RCT: Randomized controlled trial
GLP-1 RA: Glucagon-like peptide-1 receptor agonist
ADA: American Diabetes Association
BP: Blood pressure
PRISMA: Preferred reporting items for systematic reviews and meta-analyses
MACE: Major adverse cardiovascular events
eGFR: Estimated glomerular filtration rate
RR: Risk ratio
3-point MACE: MACE-3
MI: Myocardial infarction
CI: Confidence interval
