# Supplementary material for: Cardiovascular and renal outcomes with SGLT-2 inhibitors versus GLP-1 receptor agonists in patients with type 2 diabetes mellitus and chronic kidney disease: a systematic review and network meta-analysis
Source: Cardiovasc Diabetol. 2021 Jan 7;20:14. doi: 10.1186/s12933-020-01197-z (PMC7792332; doi:10.1186/s12933-020-01197-z)
Supplement: Supplementary file 1 — Additional file 1. Supplementary tables. [file 12933_2020_1197_MOESM1_ESM.docx]

Supplementary tables

Table S1

Drug subclasses of included studies.

GLP-1 RA, glucagon-like peptide-1 receptor agonists.

| Study | Drug | GLP-1 RAs subclass |
| --- | --- | --- |
| ELIXA | Lixisenatide | Exendin-4 analogue |
| EXSCEL | Exenatide | Exendin-4 analogue |
| HARMONY Outcomes | Albiglutide | GLP-1 analogue |
| LEADER | Liraglutide | GLP-1 analogue |
| PIONEER-6 | Semaglutide | GLP-1 analogue |
| REWIND | Dulaglutide | GLP-1 analogue |
| SUSTAIN-6 | Semaglutide | GLP-1 analogue |

Table S2

Prespecified definitions of renal outcomes.

SGLT2i, sodium-glucose cotransporter-2 inhibitors; GLP-1 RAs, glucagon-like peptide-1 receptor agonists; eGFR, estimated glomerular filtration rate; ESRD, end-stage renal disease; vs., versus.

| Study | Definition of renal outcomes |
| --- | --- |
| SGLT2i vs. placebo | |
| EMPA-REG OUTCOME | Macroalbuminuria, doubling creatinine, ESRD, renal death |
|  |  |
| GLP-1 RAs vs. placebo | |
| EXSCEL | Macroalbuminuria, ≥ 40% eGFR decline, ESRD, renal death |
| LEADER | Macroalbuminuria, doubling creatinine, ESRD, renal death |
| REWIND | Macroalbuminuria, ≥ 30% eGFR decline, ESRD, renal death |
